# Supplementary material for: WordCommentsAnalyzer: A windows software tool for qualitative research
Source: F1000Res. 2018 Sep 4;7:536. Originally published 2018 May 3. [Version 2] doi: 10.12688/f1000research.14819.2 (PMC6137411; doi:10.12688/f1000research.14819.2)
Supplement: Supplementary file 3 [file f1000research-7-17614-s0002.tgz › b2a79496-4ada-47c4-9682-a99ce867836d.zip!/Eight Iranian Health Journals/Archives of Rehabilitation_54.docx]

© Archives of Rehabilitation. This work is licensed under CC BY-NC 4.0 (https://creativecommons.org/licenses/by-nc/4.0/).

Volume & Issue: دوره 7 - شماره 2

Title: بررسی تأثیر تفریح‌درمانی بر مهارت‌های اجتماعی بیماران مبتلا به اسکیزوفرنیای با سیر طولانی

http://rehabilitationj.uswr.ac.ir/article-1-44-fa.html

هدف: تفریح درمانی یکی از روشهای توانبخشی می‌باشد که در این نوع درمان، درمانگر با مشارکت دادن مددجو در انواع فعالیتهای مختلف که جنبه‌ تفریحی و سرگرمی نیز دارند بدنبال دستیابی به اهدافی همچون بهبود مهارتهای اجتماعی می‌باشد. هدف این تحقیق پاسخ به این سئوال بوده است که آیا تفریح درمانی می‌تواند منجر به بهبود مهارتهای اجتماعی (رفتاری و ارتباطی) گردد؟ روش بررسی: دریک مطالعه مداخله‌ای از نوع قبل و بعد 45 مددجوی اسکیزوفرنیک با سیر طولانی با روش نمونه‌گیری مبتنی بر هدف انتخاب و سپس کلیه نمونه‌‌ها توسط ابزار اندازه گیری آسیب های وارده بر مهارتهای روزمره زندگی مورد ارزیابی قرار گرفتند، سپس مداخله تحقیق که شامل یک سفر تفریحی 10 روزه به شهر مشهد مقدس بود انجام گردید و پس از آن مجدداً کلیه نمونه‌ها توسط ابزار تحقیق مورد ارزیابی مجدد قرار گرفتند. جهت تجزیه و تحلیل داده‌‌ها، میانگین نمرات کسب شده مددجویان در ارزیابی‌های قبل و بعد با آزمون تی دانشجویی مورد بررسی قرار گرفت. یافته‌ها: از نظر آماری میانگین نمرات مهارتهای ارتباط غیرکلامی، کلامی، رفتارهای مرتبط با ابراز وجود نسبت به قبل از مداخله افزایش یافته بود که از نظر آماری معنی دار بود (P>0/001)، همچنین معیارهای مربوط به پرخاشگری و مشکلات رفتار اجتماعی نیز کاهش یافته بود که نسبت به قبل از مداخله از نظر آماری با بیش از 99 درصد اطمینان معنی دار بوده است (P>0/001 و P>0/006). نتیجه‌گیری: تفریح درمانی در بهبود وضعیت مهارتهای اجتماعی (ارتباطی و رفتاری) مددجویان اسکیزوفرنیک مؤثر است.

Title: بررسی تکرار پذیری اندازه‌گیری قدرت عضلات فلکسور و اکستانسور گردن توسط دستگاه ایزومتر 2

http://rehabilitationj.uswr.ac.ir/article-1-41-fa.html

هدف: با کمک وسیله اندازه‌گیری قدرت عضله می‌توان نیروی یک گروه عضلانی را اندازه‌گیری کرد. از طرف دیگر برای تعیین اثرات برنامه‌های درمانی و یا آموزشی به روشهایی با قابلیت تکرارپذیری صحیح و قابل استناد نیاز می‌باشد. هدف ازاین مطالعه تعیین میزان تکرارپذیری مقادیر اندازه‌گیری شده قدرت ایزومتریک عضلات گردن بوسیله دستگاه ایزومتر2 بود. روش بررسی: در یک مطالعه تحلیلی 37 فرد سالم غیر ورزشکار (18 مرد و 19 زن بین سنین 25-18سال) با انتخاب تصادفی در این مطالعه شرکت کردند. از یک Load Cell جدید که بر روی دستگاه قدیم نصب شده بود استفاده شد. قدرت ایزومتریکی عضلات فلکسور و اکستنسور گردن در زمانها و روزهای متفاوت و بکمک دو تست کننده با استفاده از وسیله جدید (دستگاه ایزومتر2) اندازه‌گیری شد.  یافته‌ها: نتایج تکرارپذیری در تست‌های Inter-rater ،test-retest inter-trial  نشان‌دهنده (N1/50/001) قدرت عضلات فلکسور و اکستنسور گردن در زنان به ترتیب 1/46% و 8/30% نسبت به مردان بود. نتیجه‌گیری: در این مطالعه ما از دستگاه جدیدتری (ایزومتر 2) نسبت به دستگاه قدیمی (ایزومتر) استفاده کردیم.. انداره گیریهای قدرت ایزومتریک عضلات گردن که بوسیله دستگاه ایزومتر2 بدست آمد بصورت یک روش مفید برای اندازه‌گیری قدرت عضلات فلکسور و اکستنسورگردن ظاهر گردید.

Title: طراحی و ساخت یک سیستم الکترونیک برای کنترل پروتز مایوالکتریک زیر آرنج

http://rehabilitationj.uswr.ac.ir/article-1-42-fa.html

هدف: هدف در این مقاله ثبت سیگنال ای.ام.جی از دو کانال مستقل از عضلات دوسر و سه سر جهت ایجاد یک سیستم کنترلی برای پروتز مایوالکتریک است. روش بررسی: به این منظور یک سیستم اخذ و پردازش سیگنال طراحی و ساخته شد. بر اساس سیگنالهای ثبت شده به کمک این دستگاه از عضلات دوسر و سه سر یک سیستم کنترل مناسب برای پروتز مایوالکتریک ایجاد گردید. یافته‌ها: بر اساس نتایج بدست آمده در این تحقیق مشخص شد که استفاده از مقدار متوسط سیگنال جهت استخراج فرمان حرکت روش بسیار موفقیت آمیزی است. درعین حال بین سرعت حرکت در دست سالم و IAV نیز رابطه‌ای خطی بدست آمد. نتیجه‌گیری: با توجه به اینکه دامنه و فرکانس سیگنال EMG کاملاً تعریف شده نیست و در بعضی زمانها فعالیتهای الکتریکی نا خواسته روی پوست وجود دارد، کنترل پروتز برای بیمار تا حدی مشکل می‌شود. همچنین در آزمون هایی که انجام گرفت مرز مشخصی بین انقباضات شدید و ضعیف در فضای IAV بدست نیامد، ولی بطور معمول مقدار متوسط سیگنال موفقترین روش جهت استخراج فرمان حرکت است.

Title: بررسی تأثیر تمرینات تعادلی برآزمونهای تعادلی و محدوده ثباتی پویا در مردان سالم با استفاده از سیستم تعادلی بایودکس

http://rehabilitationj.uswr.ac.ir/article-1-43-fa.html

هدف: علیرغم مزایای تمرینات تعادلی و نقش مهمی که می‌توانند در جلوگیری از ضایعات لیگامانی اندام تحتانی ایفا کنند،از لحاظ تحقیقی به این تمرینات توجه کافی نشده است. لذا مطالعه حاضر با هدف بررسی تأثیر تمرینات تعادلی بر تقویت لیگامانهای اندام تحتانی و آزمونهای تعادلی و محدوده ثباتی پویا در افراد سالم انجام شده است. روش بررسی: در مطالعه حاضر که به روش شبه تجربی از نوع مورد - شاهد بود گروه مورد که شامل دو گروه 17 نفری بود تمرینات تعادلی را به مدت 6 هفته انجام داد. (یک گروه روی اندام غالب و گروه دیگر روی اندام غیر غالب) گروه شاهد 17 نفری هم هیچ تمرینی انجام نداد. نمونه‌ها بصورت اتفاقی از جامعه در دسترس انتخاب شدند. قبل و بعد از انجام تمرینات دو گروه با استفاده از سیستم تعادلی بایودکس مورد ارزیابی و ارزیابی مجدد قرار گرفتند. یافته‌ها: مقایسه شاخصهای تعادلی در دو گروه نشاندهنده اختلاف معنی‌دار آماری در بعضی از آنها بود. که مهمترین آنها در شاخص‌ کلی ثباتی اندامهای غالب و غیر غالب، شاخص قدامی – خلقی اندام غیر غالب، شاخص داخلی – خارجی اندام غالب و زمان کلی آزمون محدوده ثباتی بایودکس اندام غالب بعد از انجام تمرینات تخته تعادل است (P>0/05). نتیجه‌گیری: با توجه به یافته‌های موجود می‌توان از تمرینات تعادلی به عنوان ابزاری مناسب برای جلوگیری از مشکلات لیگامانی استفاده کرد.

Title: بررسی شیوع پوسچر Sway back و تغییرات ایجادشده در وضعیت بخش‌های مختلف ستون فقرات، لگن و مفاصل اندام تحتانی در این دفورمیتی

http://rehabilitationj.uswr.ac.ir/article-1-45-fa.html

هدف: پوسچر (S.B) Sway back یکی از شایعترین انواع اختلال پوسچر معرفی شده است، اما در مورد تغییر راستای مفاصل، اطلاعات محدود و متناقضی وجود دارد. بنابراین هدف از این مطالعه، بررسی شیوع اختلال پوسچرS.B در زنان جوان و مطالعه کمّی راستای مفاصل در این پوسچر می‌باشد. روش بررسی: این مطالعه به روش .... برروی 51 نفر از دختران دانشجو بین سنین 34-19 سال که با انتخاب تصادفی ساده از بین دختران دانشگاه علوم پزشکی مشهد انتخاب شده بودند انجام شد. به همین منظور، با استفاده از صفحه شطرنجی و امتداد شاقولی، میزان نوسان (جابجایی خطی) لگن اندازه‌گیری شد و پوسچر افرادیکه خط شاقولی با فاصله بیش از cm3 از خلف نقطه میانی محور واسطASIS و PSIS عبور می‌کرد بعنوان پوسچر S.B شناسایی شد. سپس مقدار شیب لگن به کمک شیب سنج لگنی و زاویه کایفوز و لوردوز توسط خط کش قابل انعطاف و راستای مفاصل مچ، زانو و هیپ به وسیله گونیامتر اندازه‌گیری شد. یافته‌ها: میانگین لوردوز افراد در پوسچر S.B بیش از افراد نرمال بود (0/004=P) و میزان کایفوز و تیلت قدامی لگن در افراد این گروه گرچه بیش از افراد گروه نرمال بود ولی از نظر آماری تفاوت معنی‌داری نداشت (P>0/05). همچنین مفاصل ران و زانو در پوسچر S.B به سمت فلکسیون سوق پیدا کرده بود و مچ پا دورسی فلکسیون نشان می‌داد که مقدار دورسی فلکسیون با میزان نوسان لگن ارتباط معنی‌دار مستقیم داشت. شیوع این دفورمیتی در افراد مورد مطالعه %39/2 و بیش از دفورمیتیهای دیگر ستون فقرات بود. نتیجه‌گیری: مشاهدات این تحقیق نشان داد که S.B شایعترین دفورمیتی ستون فقرات در زنان جوان جامعه ماست. همچنین نشان داد که با وجود افزایش لوردوز، امتداد مفاصل اندام تحتانی، ناحیه توراسیک و شیب (تیلت) لگن نسبتاً طبیعی باقی می‌ماند.

Title: مقایسه مهارتهای حرکتی دانش آموزان کلاس اول دختر و پسر منطقه 3 آموزش و پرورش استان تهران

http://rehabilitationj.uswr.ac.ir/article-1-46-fa.html

هدف: این پژوهش تحقیقی است توصیفی ـ تحلیلی که به منظور یافتن تفاوتهای مهارتهای حرکتی دختران و پسران 7 ساله در سال 83 به انجام رسیده است. روش بررسی: جامعه مورد نظر شامل 120 نفر (60 دختر و 60 پسر) می‌باشد که از 6 دبستان از منطقه 3 آموزش و پرورش انتخاب شده‌اند. نمونه گیری به روش تصادفی ساده انجام شده است. ابزار تحقیق شامل مقیاس رشد حرکتی لینکلن اوزورتسکی و پرسشنامه مربوط به اطلاعات شخصی می‌باشد. بعد از انجام ارزیابیهای انفرادی و جمع آوری داده‌ها، کلیه اطلاعات با استفاده از نرم افزارspss و آزمون sample T _ Test تجزیه و تحلیل گردیده و مقایسه میانگین نمرات مهارتهای حرکتی انجام گرفت. یافته‌ها: یافته‌های حاصل در ارتباط با اهداف پژوهش نشان دادند که بین میانگین نمرات مهارتهای حرکتی کلی، تعادل کلی، تعادل با چشم باز، تعادل با چشم بسته، تعادل داینامیک و استاتیک با چشم باز، هماهنگی حرکتی دو طرفه، هماهنگی اندامهای فوقانی، هماهنگی اندامهای فوقانی ـ تحتانی و دقت وسرعت حرکتی در دو گروه دختران و پسران کلاس اول ابتدایی تفاوت معنی داری وجود ندارد. تنها تفاوت معنی‌دار بین میانگین نمرات هماهنگی چشم و دست در دو گروه دختران و پسران می‌باشد. نتیجه‌گیری: کاربرد نتایج این پژوهش در حیطه‌های مختلف درمانی ـ آموزشی رشد روانی حرکتی کودکان می‌باشد و می‌توان از این یافته‌ها در جهت بهبود هرچه بیشتر برنامه‌ریزی‌های مرتبط با کودکان در مراکز مختلف دولتی و غیر دولتی بهره برد.

Title: دختران و زنان دچار معلولیت جسمی -حرکتی: نیازها و مشکلات

http://rehabilitationj.uswr.ac.ir/article-1-47-fa.html

هدف: پژوهش حاضر، با این رویکرد که پاسخگویی مناسب به نیازها و مشکلات دختران و زنان معلول، مستلزم شناسایی اولیه این نیازها و مشکلات است، هدف اصلی خود را تعیین نیازها و مشکلات دختران و زنان دچار معلولیت‎ جسمی - حرکتی، در شهرستان تهران قرار داد. روش بررسی: نوع این مطالعه توصیفی است. نمونه‎ی مورد بررسی 216 دختر و زن دارای معلولیت هستند که با روش انتخاب تصادفی منظم از بین 1395 نفر مراجعه کنندگان مرکز بهزیستی شهرستان تهران انتخاب شدند. جمع‎آوری اطلاعات با استفاده از روش مصاحبه و ابزار پرسشنامه محقق ساخته‎ی 82 سؤالی انجام شد. پرسشنامه بر اساس مرور منابع و نتایج جلسات بحث گروهی متمرکز تنظیم گردید و پس از تعیین روایی محتوی و پایایی آن (با آلفا کرونباخ 0/76) به اجرا در آمد. یافته‌ها: بررسی نیازها و مشکلات دختران و زنان دارای معلولیت جسمی - حرکتی، به طور کلی اولویت‎های آنان را در هر زمینه مشخص کرد. اولویت نیاز آموزشی. آموزش جامعه و آموزش حرفه‌ای. نیاز اشتغال: سرویس رفت و آمد و وجود سهیمه‎ خاص برای اشتغال. نیاز به تشکیل خانواده: فراهم آمدن امکان شناخت بیشتر همسر آینده توسط خانواده؛ مشاوره‎ی قبل از ازدواج، رفت و آمد: مناسب‎سازی پیاده‎روها و معابر عمومی، وجود سرویس رفت‎وآمد خاص متناسب با نوع معلولیت؛ نیاز به خدمات توانبخشی: نیاز به وسایل کمک توانبخشی و خدمات آموزشی؛ گذراندن اوقات فراغت: کمکهای مالی برای استفاده از امکانات ورزشی - تفریحی، وجود مکانهای ورزشی متناسب معلولیت آنان؛ برقراری ارتباط: آگاهی دیگران به توانایی‎های افراد معلول در کنار محدودیت، برخورد عادی و بدون ترحم از سوی دیگران و همراهی یکی از نزدیکان هنگام حضور در جمع. اولویت مشکلات در زمینه آموزش: جابجایی و رفت‎وآمد، در دسترس نبودن منابع و امکانات آموزشی؛ اشتغال: جابجایی و رفت و آمد، عدم وجود ضمانتهای اجرایی و حمایت از اشتغال معلولان. مشکل زندگی مشترک: دخالت اطرافیان، خشونت همسر، انجام امور خانه‌داری. خشونت: بی‌توجهی، رفتار پرخاشجویانه رفت و آمد: نامناسب بودن وسایل نقلیه عمومی با توجه به شرایط و نیازهای فرد، نامناسب بودن پیاده‌روها و معابر عمومی. مشکل گذراندن اوقات فراغت: نبودن سرویس رفت و آمد خاص برای استفاده از مکانهای تفریحی - ورزشی، مشکل مالی برای استفاده از امکانات از این مکانها. نتیجه‌گیری: نتیجه‌ نهایی حاصل از این پژوهش، نه تنها نشان دهنده تنوع مشکلات و نیازهای دختران و زنان دچار معلولیت جسمی – حرکتی در جامعه مورد مطالعه است، بلکه تفاوت اولویت این نیازها و مشکلات را در زمینه‌های آموزش، اشتغال، ازدواج و تشکیل خانواده، رفت و آمد، درمان و توانبخشی، اوقات فراغت، برقراری ارتباط با دیگران، از دیدگاه خود این افراد مشخص می‌کند.

Title: مدیریت ارائه خدمات سلامتی در زمان وقوع بلایای طبیعی

http://rehabilitationj.uswr.ac.ir/article-1-48-fa.html

هدف: مدیریت مؤثر سلامتی در بلایا بستگی به پیش بینی و شناسایی مشکلات حاصل از بلایا و در نظر گرفتن امکانات لازم در زمان و مکان مناسب و مقتضی دارد. در این مقاله که قسمتی از یک مطالعه بزرگتر می‌باشد سعی شده است بر اساس تجربیات ارائه دهندگان خدمات سلامتی در بلایا عوامل مؤثر در مدیریت خدمات سلامتی در زمان وقوع بلایا بررسی شود. روش بررسی: پژوهش حاضر به روش گراندد تئوری انجام شده است. مشارکت کنندگان 16 نفر شامل 4 نفر پرستار ،2 نفر روانپزشک، 1 نفر اپیدمیولوژیست، 1 نفر مددکار، 1 نفر روانشناس، یک نفر متخصص بهداشت،1 نفر کارشناس ارشد بهداشت،1 نفر بهیار، 3 نفر از اهالی بم ویک نفر پزشک عمومی بودند. از کل مشارکت کنندگان 14 نفر مرد و2 نفر زن و میانگین سنی آنها 37 سال بود. کلیه مشارکت کنندگان حداقل تجربه حضود در یک بلیه را داشتند. برای جمع آوری داده ها از مصاحبه های نیمه ساختارمند استفاده شد. کلیه مصاحبه ها ضبط و سپس کلمه به کلمه دست نویس شده وبا استفاده از روش استراوس وکوربین تجزیه و تحلیل شدند. یافته‌ها: مسئله مدیریت در ارائه خدمات سلامتی مورد تأکید مشارکت کنندگان در مطالعه قرار داشت و در ارتباط با این مفهوم مهم برنامه ریزی، سازماندهی، هماهنگی و مشارکت سایر کشورها مطرح شد. نبود برنامه ونظم در ارائه خدمات سلامتی، نبود تقسیم کار، مشخص نبودن وظایف، تداخل کار وهمپوشانی، موازی کاری، عدم هماهنگی بین مدیران وارائه دهندگان خدمات، خود محوری، ناتوانی سازمان ملل در هماهنگی و جلب مشارکتهای بین المللی موانع اصلی تأمین خدمات سلامتی مورد نیاز بازماندگان در زمان وقوع بلایاست ومدیریت مناسب با توجه به برنامه ریزی و کسب آمادگی قبلی برمبنای تجارب قبلی و اطلاعات حاصل از بررسی منطقه، برنامه ریزی و تصمیم گیری برمبنای اطلاعات، تأمین نیروی انسانی کافی، تقسیم کار صحیح و سریع، برقراری ارتباط از بدو ورود، هماهنگی از ابتدا درون و بین گروهها و استفاده از اطلاعات برای انجام هماهنگی، برخورد مناسب و قدردانی از خدمات گروههای خارجی و برقراری ارتباط مناسب با آنها می‌تواند خدمات سلامتی را در زمان وقوع بلایا تسهیل کند. نتیجه‌گیری: با توجه به اینکه مسئله مدیریت در ارائه خدمات سلامتی مورد تأکید شرکت کنندگان قرار داشته و از طرفی مدیریت مناسب نقش مهمی در ارائه خدمات سلامتی هماهنگ، منسجم، دردسترس و مستمر دارد، در نتیجه کسب آمادگی از قبل، توجه به اهمیت برنامه ریزی ملی، استانی و محلی، تأمین نیروی انسانی کافی و تقسیم کار صحیح و سریع برای ارائه خدمات سلامتی، هماهنگی نیروها و تجهیزات، انجام هماهنگی توسط ستاد مرکزی بلایا واقع در محل به همراه جلب مشارکت بین المللی با برخورد مناسب زمینه را برای ارائه مدیریت مناسب خدمات سلامتی در بلایا فراهم می‌کند.

Title: مقا‌یسه‌ حافظه کوتاه‌مدت بیماران مالتیپل اسکلروزیس با افراد طبیعی

http://rehabilitationj.uswr.ac.ir/article-1-49-fa.html

هدف: مالتیپل اسکلروزیس یک بیماری سیستم عصبی مرکزی است. الگوی اصلی آسیب عصب روانشناختی در بیماران ام.اس با نقصهای حافظه و توجه مشخص می‌شود. مشکل حافظه تقریباً در 50 تا 60 درصد افراد ام.اس اتفاق می‌افتد. هدف مطالعه حاضر، بررسی عملکرد حافظه در بیماران ام.اس می‌باشد. روش بررسی: 40 بیمار مالتیپل اسکلروزیس، (30 بیمار تشدید شونده – بهبود یابنده «نوع عود و فروکش» و 10 بیمار نوع پیشرونده مزمن)، با استفاده از مقیاس حافظه لوریا-نبراسکا ارزیابی شدند. یافته‌ها: همه بیماران ام. اس (بدون افسردگی شدید که با مقیاس افسردگی بک بررسی شد) آسیب معنی‌داری در تمام متغیرهای حافظه (کلامی و غیر کلامی، تأخیری و عملکرد کلی حافظه) نسبت به گروه کنترل نشان دادند. تفاوت عملکردهای حافظه در بین بیماران با دو نوع ام. اس معنی‌دار نبود. علاوه بر این بین ضعف حافظه و آسیبهای ام.آر.آی تفاوت معنی‌داری وجود نداشت. نتیجه‌گیری: این مطالعه از این جهت که منتهی به فهم بهتر نقصهای حافظه در بیماران ام.اس می‌شود، اهمیت دارد. علاوه بر این راهبردهای خاص توانبخشی براساس الگوهای آسیب حافظه را طرح ریزی می‌نماید.

Title: بررسی تأثیر کاربرد شیوه ایمن‌سازی در مقابل استرس بر میزان استرس پرستاران

http://rehabilitationj.uswr.ac.ir/article-1-50-fa.html

هدف: استرس پدیده‌ای پیچیده و فردی و جزء لاینفک زندگی بشر است که گاهی مشکل ساز می‌شود و اگر انسان قادر به سازگاری با آن نباشد جسم و روان او مورد تهدید قرار می‌گیرد. تمایل به کنار آمدن با استرس راه مثبتی است که باعث دستیابی تدریجی به ارتقاء سلامتی می‌شود. بنابراین پژوهش حاضر با هدف تعیین تأثیر کاربرد شیوه ایمن‌سازی در مقابل استرس بر میزان استرس پرستاران شاغل در مراکز آموزشی درمانی قم انجام شده است.  روش بررسی: پژوهش حاضربه صورت مطالعه نیمه تجربی بوده و جامعه پژوهش شامل 200 نفر پرستار بودند که از بین آنها 60 نفر با روش نمونه‌گیری تصادفی ساده انتخاب و در گروه آزمون و شاهد (گروه 30 نفری) قرار گرفتند. ابزار پژوهش پرسشنامه سنجش استرس دروگاتیس بود. کلیه آزمودنیهای تحقیق قبل از مداخله با ابزار فوق‌الذکر سنجش شدند. سپس مداخله که شامل یک کارگاه دو روزه ایمن‌سازی در مقابل استرس بود برای گروه آزمون اجرا شد. پس از پایان مداخله مجدداَ دو گروه با پرسشنامه سنجش استرس دروگاتیس تست شدند و نتایج قبل و بعد دو گروه با آزمون تی زوجی و مستقل مورد بررسی قرار گرفت. یافته‌ها: نتایج نشان داد که میانگین میزان استرس در بعد محیطی، شخصیتی و هیجانی و مجموع سه بعد، قبل از مداخله از نظر آماری بین دو گروه با هم تفاوت معنی‌دار ندارد ولی بعد از مداخله این اختلاف از نظر آماری معنی‌دار می‌باشد (P>0/05). نتیجه‌گیری: اجرای شیوه ایمن‌سازی در مقابل استرس بر کاهش استرس پرستاران مؤثر بوده و می‌تواند در جهت بهبود کیفیت مراقبتها و همچنین ارتقاء سلامتی و کنترل استرس بکار رود.

Title: بررسی میزان آگاهی خانم‌ها نسبت به معاینه پستان توسط خودشان در مراجعه کنندگان به درمانگاه زنان بیمارستان‌های نجمیه و بقیه‌الله(عج)

http://rehabilitationj.uswr.ac.ir/article-1-51-fa.html

هدف: شیوع سرطان پستان در 50 سال اخیر رو به افزایش بوده است و در ایران بروز آن یک دهه از کشورهای پیشرفته زودتر مشاهده شده است. داشتن آگاهی و شرکت در غربالگری سرطان پستان پیش آگهی آن را بهتر می‌کند. هدف ما در این مطالعه ارزیابی میزان آگاهی خانمها نسبت به معاینه پستان توسط خود (SBE) بوده است. روش بررسی: در یک مطالعه توصیفی – مقطعی 376 خانم بین 70-12 سال را که به درمانگاه‌های زنان بیمارستان نجمیه و بقیه‌الله(عج) مراجعه کرده بودند بصورت سرشماری انتخاب کردیم و پرسشنامه ای را که به همین منظور تهیه کرده بودیم را به آنها تحویل و سپس جمع آوری نمودیم و پس از کدبندی با برنامه کامپیوتری SPSS مورد تجزیه و تحلیل آماری قرار دادیم. یافته‌ها: از 376 نفر جمعیت مورد مطالعه ما 243 نفر (%6/64) اظهار داشته‌اند که نسبت به چگونگی انجام SBE آگاهی دارند و در مجموع 186 نفر (%4/49) آنرا انجام داده اند که 51 نفر (%4/27) منظم و 44 نفر (%7/23) به موقع و فقظ 10 نفر (%3) هم منظم و هم به موقع معاینه را انجام داده اند. نتیجه‌گیری: در جمعیت مورد مطالعه ما یا آگاهی نسبت به SBE وجود ندارد و یا آن را به موقع و منظم انجام نمی دهند و لذا آموزش چگونگی و اهمیت انجام SBE جهت نیل به اهداف پیشگیرانه و بهبود درمان در مراحل اولیه و ارتقاء بهزیستی مبتلایان توصیه می‌شود.

Title: بررسی تأثیر دهان‌شویه گل بابونه بر پیشگیری از استوماتیت ناشی از شیمی‌درمانی

http://rehabilitationj.uswr.ac.ir/article-1-52-fa.html

هدف: استوماتیت یا موکوزیت دهانی یکی از مشکلات اساسی در بیماران تحت شیمی درمانی می‌باشد. این عارضه جانبی تقریباٌ در 40 تا 100 درصد بیماران بروز می‌کند. استوماتیت دردناک بوده و ممکن است با دریافت غذا و مایعات و همچنین زندگی روزمره بیمار تداخل کند. در فرد سرطانی این حالت ممکن است سبب افزایش خطر عفونت، بستری شدن طولانی مدت و مرگ و میر شده و درمان سرطان را به تأخیر اندازد. هدف از این تحقیق تعیین میزان تأثیر دهانشویه گل بابونه بر پیشگیری از استوماتیت ناشی از شیمی درمانی می‌باشد. روش بررسی: این پژوهش از نوع کارآزمایی بالینی دوسو کور می‌باشد که به منظور بررسی تأثیر دهانشویه گل بابونه بر استوماتیت ناشی از شیمی درمانی در مرکز انکولوژی شهید قاضی طباطبایی تبریز انجام شده است. در این تحقیق 28 بیمار درگروه آزمون و14 بیمار در گروه شاهد به صورت تصادفی قرار داده شدند. بعد به بیماران گروه آزمون دهانشویه تهیه از گل بابونه داده شد تا روزانه چهار بار دهان خود را به مدت 16 روز شستشو دهند. مخاط دهان بیماران روزانه توسط دو نفر همکار محقق که از نوع دهانشویه بی اطلاع بودند بررسی می‌شد. یافته‌ها: نتایج بدست آمده نشانگر آن است که دهانشویه گل بابونه به طور معنی‌داری توانسته است در پیشگیری از استوماتیت ناشی از شیمی درمانی( شدت استوماتیت، شدت درد و میزان بقای استوماتیت) مؤثر باشد (P<0/05). نتیجه‌گیری: از آنجایی که تنها و بهترین راه پیشگیری استوماتیت ناشی از شیمی درمانی، بکارگیری دهانشویه مناسب می‌باشد، بنابراین استفاده از دهانشویه گل بابونه می‌تواند در پیشگیری از استوماتیت این بیماران مؤثر بوده و کیفیت درمان و کیفیت زندگی بیماران شیمی درمانی را بهبود بخشد.

Title: کشف بیماری آلزایمر

http://rehabilitationj.uswr.ac.ir/article-1-53-fa.html

کرپلین در هشتمین ویرایش (1910) از: «روانپزشکی، کتاب درسی برای دانشجویان و پزشکان» چنین نوشت که «گروه ویژه‌ای از موارد با دگرگونیهای یاخته‌ای بسیار شدید» به وسیله آلزهایمر مورد بحث قرار گرفته است. آن دگرگونیها عبارت بودند از: «پلاکهای بیش از حد متعدد، مرگ تقریباً یک سوم از یاخته‌های قشر مغز، که به عوض آنها کلافه‌های خاصی از نوروفیبریلها با رنگ تند جایگزین شده بودند». طبق نظر کرپلین، این دگرگونیها «نشان دهنده شدیدترین شکل خردزدودگی سالمندی است». کرپلین هنگامیکه نوشت: «تعبیر بالینی این بیماری آلزهایمر هنوز نامعلوم است»، در واقع برای نخستین بار به عنوان «بیماری آلزهایمر» اشاره کرد. از همان زمان ابهاماتی درباره تشخیص بیماری آگوسته دتر (نخستین موردی که آلزهایمر در 1906 معرفی کرد)، و دلیل نامیدن این وضعیت با عنوان «بیماری آلزهایمر» توسط کرپلین وجود داشت. اکنون پس از یکصد سال به نظر می‌رسد پاسخهای معتبری به این پرسشها در دست است. توصیف خردزدودگی آگوسته دتر به وسیله آلزهایمر در 1907، و سپس به وسیله پروسینی در 1909، به پلاکهای پیری و کلافه‌های نوروفیبریلری اشاره داشت. افزون براین، پروسینی مغز این بیمار را بازبینی کرد و هیچ نشانه چشمگیری از آرتریواسکلروزیس که برخی به عنوان تشخیص این بیمار به آن معتقد بودند نیافت. در سال 1998 دانشمندان در بخش نوروبیولوژی انستیتوی ماکس پلانک در مارتینز رید آلمان، و در دانشگاه مونیخ برشهای مغزی نخستین مورد گزارش شده از بیماری آلزهایمر را بازیافتند. بررسی این برشهای مغزی نشان دهنده شمار زیادی از کلافه‌های نوروفیبریلری و پلاکهای آمیلویید بود. بنابراین، نخستین مورد گزارش شده، نمونه دقیقی از بیماری آلزهایمر مطابق معیارهای امروزی است. این نیز جالب است که در 1997 دکتر گربر و همکارانش در قسمت روانپزشکی انستیتوی ماکس پلانک، گروه نورومرفولژی در مارتینزرید آلمان، برشهای مغزی بافت شناختی دومین مورد آلزهایمر به نام یوهان اف. را که با وجود گذشت نود سال به خوبی حفظ شده بود شناسایی کردند. بررسی این برشها پلاکهای متعدد آمیلوییدی را آشکار کرد اما هیچ کلافه نوروفیبریلری در قشر مغز یافت نشد. این وضعیت با شکلی کمتر شایع از بیماری آلزهایمر مطابق است که می‌توان عنوان «فقط پلاک» را به آن داد. پژوهشگران در انستیتوی ماکس پلانک غربالگری موتاسیونی اکسون 17 از پیش ساز آمیلوییدی ژن پروتئین، و ژنوتیپینگ برای آلل‌های E آپولیپوپروتئین انجام دادند. معلوم شد که این بیماری برای آلل E اپسیلون 3 آپولیپوپروتئین، هموزیگوت است و فاقد موتاسیونهای APP در کودونهای 692، 693، 713 و 717 می‌باشد. در مورد برشهای مغزی آگوسته دتر، ژنوتیپینگ برای آلل های E اپسیلون 4 آپولیپوپروتئین، فقدان این عامل خطر برای بیماری آلزهایمر را نشان داد و در عین حال معلوم کرد که تحلیل موتاسیونی بافتهای مغزی به جا مانده از نود سال پیش هنوز عملی است. به این ترتیب در یکصدمین سالگرد کشف تاریخی دکتر آلزهایمر، یافته‌های او دیگربار تأیید می‌شود.
